# Graphene-Based Flexible and Transparent Tunable Capacitors

**DOI:** 10.1186/s11671-015-0974-4

**Published:** 2015-07-03

**Authors:** Baoyuan Man, Shicai Xu, Shouzheng Jiang, Aihua Liu, Shoubao Gao, Chao Zhang, Hengwei Qiu, Zhen Li

**Affiliations:** College of Physics and Electronics, Shandong Normal University, Jinan, 250014 People’s Republic of China; College of Physics and Electronic Information, Dezhou University, Dezhou, 253023 People’s Republic of China; State Key Laboratory of Crystal Material, Shandong University, Jinan, 250100 People’s Republic of China

**Keywords:** Graphene, CVD, LMBE, BMN, Transparent and flexible capacitor

## Abstract

We report a kind of electric field tunable transparent and flexible capacitor with the structure of graphene-Bi_1.5_MgNb_1.5_O_7_ (BMN)-graphene. The graphene films with low sheet resistance were grown by chemical vapor deposition. The BMN thin films were fabricated on graphene by using laser molecular beam epitaxy technology. Compared to BMN films grown on Au, the samples on graphene substrates show better quality in terms of crystallinity, surface morphology, leakage current, and loss tangent. By transferring another graphene layer, we fabricated flexible and transparent capacitors with the structure of graphene-BMN-graphene. The capacitors show a large dielectric constant of 113 with high dielectric tunability of ~40.7 % at a bias field of 1.0 MV/cm. Also, the capacitor can work stably in the high bending condition with curvature radii as low as 10 mm. This flexible film capacitor has a high optical transparency of ~90 % in the visible light region, demonstrating their potential application for a wide range of flexible electronic devices.

**Electronic supplementary material:**

The online version of this article (doi:10.1186/s11671-015-0974-4) contains supplementary material, which is available to authorized users.

## Background

Dielectric thin films with voltage tunable permittivity have attracted much attention for applications in the electrically tunable microwave capacitor devices such as resonators, phase shifters, filters and antennas [1–4]. Due to low dielectric loss and large tenability, Bi_1.5_MgNb_1.5_O_7_ (BMN) film has been regarded as a promising potential candidate for tunable microwave devices [4, 5]. Using Pt or Au as electrodes, high dielectric tunability was achieved by using metal-insulator-metal (MIM) structures. However, the metal electrodes were not transparent, and the high melting temperature of Pt or Au makes them difficult to integrate with the flexible organic substrate. Thus, there is a clear and urgent need for finding a kind of transparent and flexible conductive material to combine dielectric thin films with flexible substrates. It is an important issue for the emerging flexible electronics.

The emergence of graphene brought new prospects for flexible electronic devices. Graphene has outstanding optical transmittance and high conductivity [6, 7]. It is also regarded as a “plastic electronic” material due to its high mechanical strength and excellent flexibility [8, 9]. To date, the solar cells [10], touch sensors [11], and film loudspeakers [12, 13] have been achieved by using graphene as the transparent conductive electrode. Very recently, the nanocapacitor system was fabricated by using graphene as electrodes, and a significant increase in capacitance below a thickness of ~5 nm was found [14]. The flexible graphene paper was also applied in transparent and stretchable supercapacitors and displayed very huge capacitance of 3.3 mF cm^−2^ and high capacity retention of 95.4 % [15]. These results indicate that graphene is potentially useful for diverse future electronic applications.

In this paper, we grow large-area graphene film on ~50 μm Cu foil by chemical vapor deposition (CVD) method. The BMN thin films were deposited on the graphene/Cu foil by laser molecular beam epitaxy (LMBE) technology. By transferring another graphene layer, we fabricated the capacitor with the form of graphene-insulator-graphene on Cu foil. After removing the Cu foil with FeCl_3_ etchant, we obtained a flexible and transparent tunable capacitor. The capacitor shows the largest dielectric tunability and can work stably in the high bending condition. The capacitor also has relatively high optical transparency in the visible light region, raising the freedom of design of optoelectronic devices.

## Methods

### Preparation of BMN Ceramic Targets

The BMN ceramic targets were prepared by solid state reaction process with Bi_2_O_3_, MgO, and Nb_2_O_5_ as starting materials. The mixture of starting materials in stoichiometry was ball milled for 20 h and sintered into disks in a sealed crucible at 1100 °C for 3 h.

### Growth of BMN Film on Graphene/Cu Substrate

Large-area high-quality monolayer graphene was grown on 25-μm-thick copper foils using a CVD method. The detailed growing process was reported in our previous report [12]. The BMN thin films were deposited on graphene/Cu foil using LMBE method [16]. The growing ambient was a mixture of Ar and O_2_ with the O_2_/Ar mixing ratio of 1:5. The total growing pressure was 5 Pa. During deposition, the surface temperature of the substrates was 550–800 °C, and laser fluence was 0.5 J/cm^2^. The film thickness was monitored by SQM 180 thickness gauge and was controlled to ~220 nm. The whole deposition process was 155 min with a very low growing rate of ~1.4 nm/min.

### Transfer Graphene Layer to BMN Substrate

Transfer of graphene onto BMN/graphene/Cu was performed by a wet transfer technique. A thin polymethyl methacrylate (PMMA) film (~100 nm thick) was spin-coated onto the graphene/Cu foil to support the graphene. The copper foil was etched away by 0.5 M aqueous FeCl_3_ solution. After removing the residual etchant by deionized water, the BMN/graphene/Cu substrate was placed in water with a tilting angle of ~30° underneath the floating film. Water was pulled out with a syringe to lower the film onto the substrate. After drying it under vacuum for several hours, we employed a small amount of PMMA to introduce a second PMMA coating on the precoated PMMA/graphene/BMN/Cu substrate. The redissolution of the precoated PMMA tends to mechanically relax the underlying graphene, leading to a better contact with the surface morphology of the underlying substrate. Then, the sample was heated at 170 °C in air for over 30 min to increase the adhesion between the graphene and the substrate. Finally, the PMMA was removed with an acetone bath, forming the parallel plate capacitor on Cu foil with the structure of graphene-BMN-graphene.

### Preparation Processes of the Flexible and Transparent Tunable Capacitor

**Fig 1:**
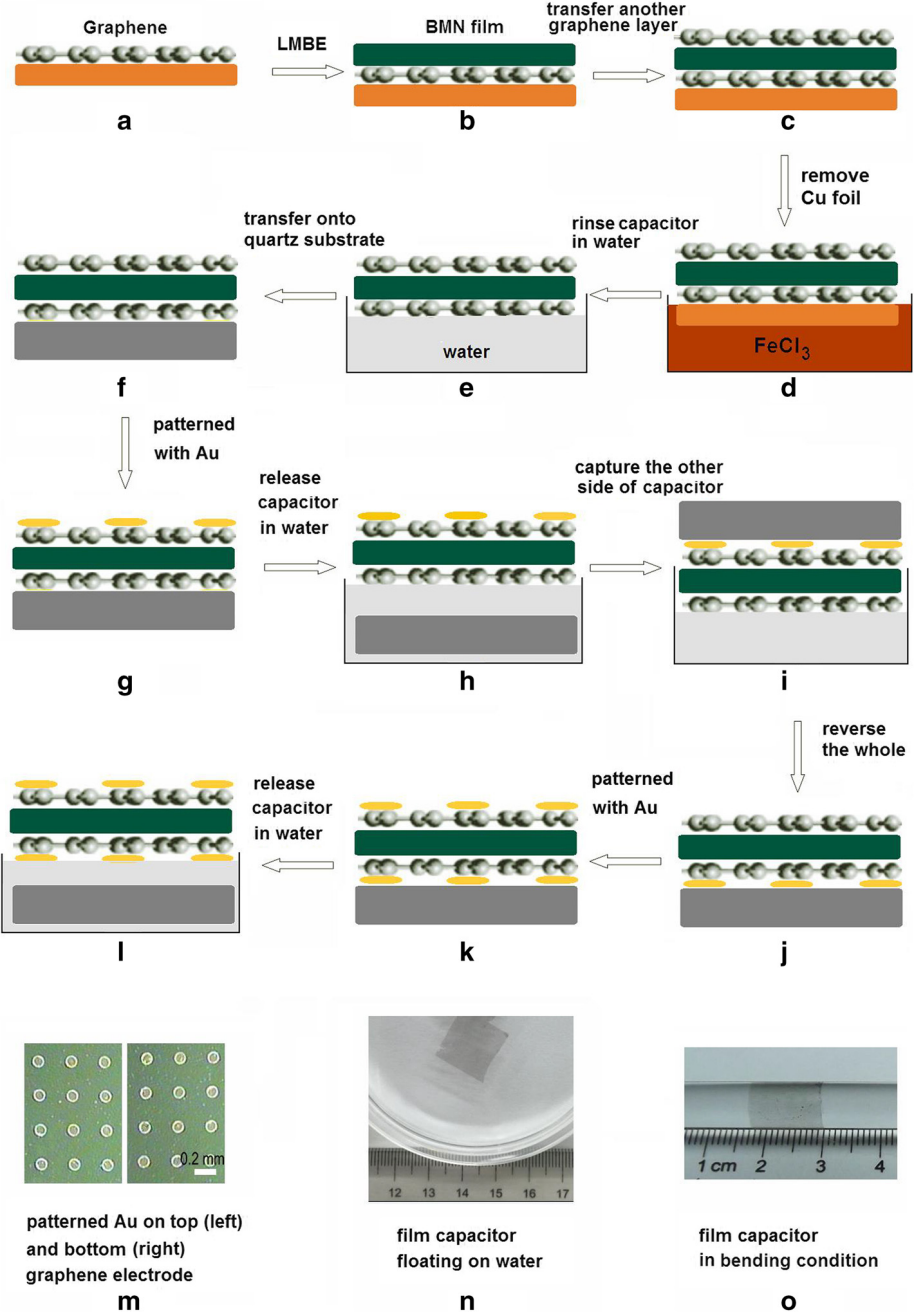
Schematic of preparation processes of the flexible and transparent tunable capacitor. **a** Graphene growth on Cu foil by CVD. **b** BMN film grown on graphene/Cu substrate by LMBE. **c** Another graphene layer transferred on BMN/graphene/Cu substrate. **d** Removal of Cu foil. **e** Removal of etchant residue in distilled water. **f** Transfer of the capacitor onto the quartz substrate. **g** Capacitor patterned with Au electrode. **h** Release of capacitor in water. **i** Capture of the other side of capacitor by quartz substrate. **j** Reversal of quartz substrate/capacitor. **k** Patterned with Au electrode on the other side capacitor. **l** Release of the patterned capacitor in water. **m** Optical micrographs of patterned Au on the *top* (*left*) and (*bottom*) graphene electrode. **n** Film capacitor floating on water. **o** The transparent capacitor around a fused silica rod

Figure 1 shows the schematic of preparation process of the transparent tunable capacitor. The graphene films were grown on the copper foil by CVD method (Fig. 1a). Using LMBE method, the BMN thin films were deposited on graphene/Cu foil (Fig. 1b). The BMN ceramic targets used in LMBE process were prepared by solid state reaction (“Methods”). By transferring another graphene layer as top electrode, we fabricated the parallel plate capacitor on Cu foil with the structure of graphene-insulator-graphene (Fig. 1c). Then, the Cu foil was removed with FeCl_3_ etchant, forming the flexible and transparent capacitor (Fig. 1d). Due to the surface tension of the water, the film capacitor was floated on the water. A flat quartz substrate was placed just under the floating the film capacitor to carefully move the film capacitor to distilled water several times to rinse the etchant residue (Fig. 1e). After the etchant residue was completely removed, the film capacitor was caught again and lay flat on the quartz substrate (Fig. 1f). After drying it under vacuum for 30 min, the Au film with 0.2 mm in diameter was fabricated on the top graphene electrode by lithography technology for measurement (Fig. 1g). Then, the capacitor/quartz was immersed into distilled water for 15 min, making the capacitor leave the quartz substrate and float on the water again (Fig. 1h). Then, the floating film capacitor was caught again by the quartz substrate to keep Au-patterned graphene contacting with substrate surface (Fig. 1i). The capacitor was carefully moved out from the distilled water and dried under vacuum (Fig. 1j). The lithography technology was carried out again to pattern Au film on the other side of capacitor (Fig. 1k). Finally, the capacitor was floated on the water by being immersed into distilled water (Fig. 1l). Figure 1m shows the optical micrographs of patterned Au film on the top (left) and bottom (right) of graphene electrode, respectively. The well patterned Au in regular arrays was formed on the BMN film for measurement. The film capacitor cannot support itself, but it can float on the water or exist by attaching on other surfaces (Fig. 1n). Figure 1o shows the capacitor around a fused silica rod, displaying good flexibility and transparency.

### Apparatus and Characterizations

The high resolution transmission electron microscopy (HRTEM) images were obtained using a transmission electron microscopy system (JEOL, JEM-2100). The quality of graphene was evaluated by using Raman spectroscopy (Horiba HR-800) with laser excitation at 532 nm (2.33 eV). The crystal structure of BMN films was characterized by X-ray diffraction (XRD, Rigaku D/MAX-RB). Surface morphologies of the samples were observed using SEM (ZEISS, SUPRATM-55) with accelerating voltage below 1 kV. The AFM system used was a Bruker Multimode 8 and was operated in noncontact mode. The transmittance of the films was obtained on a spectrophotometer (HitaChi U-4000). The sheet resistance of graphene was measured by using a semiconductor parametric analyzer (Keithley 4200-SCS). The dielectric properties of the capacitor were performed on a precision impedance analyzer (Agilent 4294A).

## Results and Discussion

### Characterizations of CVD Grown Graphene

**Fig 2:**
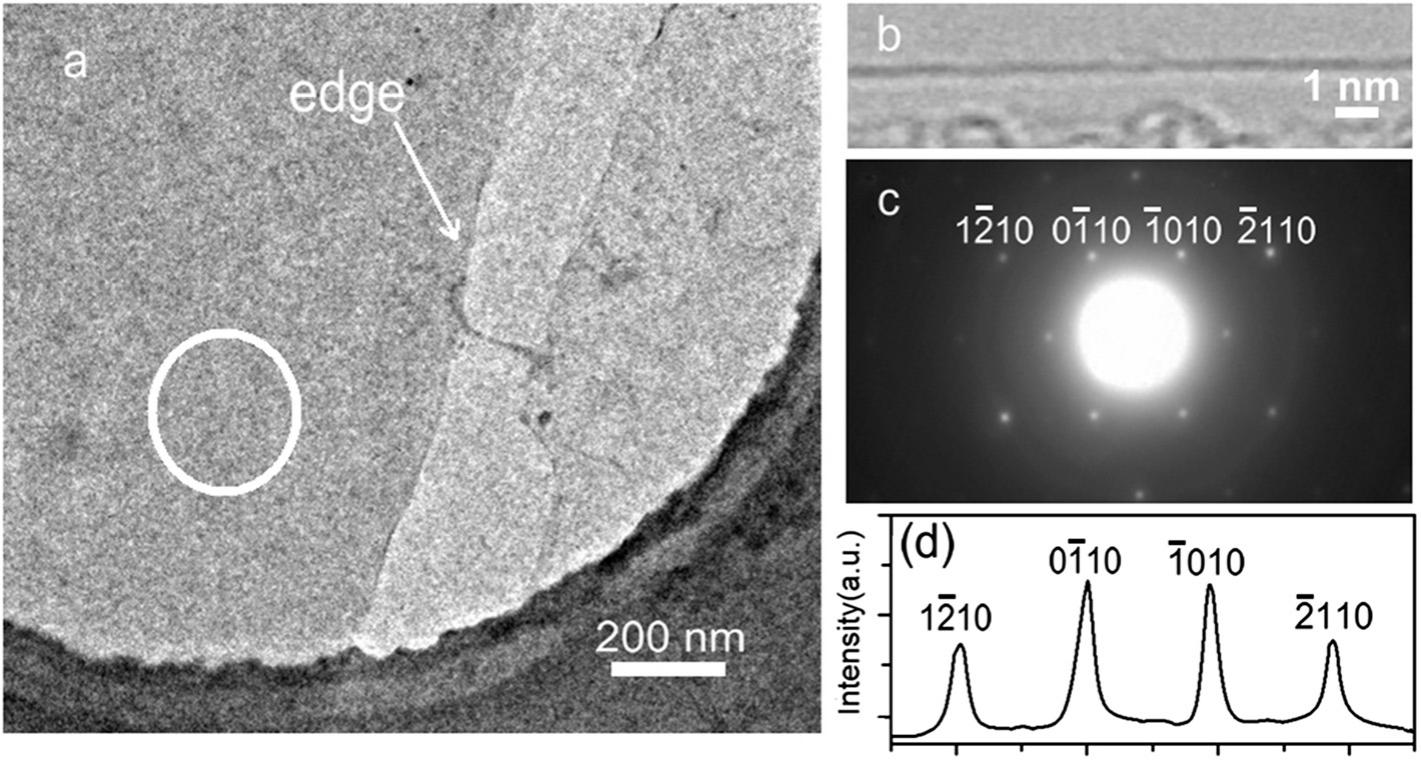
HRTEM analysis of CVD grown graphene. **a** Bright-field TEM image of a suspended graphene membrane. **b** Image of folded edge for monolayer graphene. **c** Electron diffraction patterns from the graphene membrane in (**a**). **d** Diffracted intensity taken along the $1\overset{-}{2}10$ to $\overset{-}{2}110$ axis for the patterns shown in (**c**)

HRTEM and selected area electron diffraction (SAED) were carried out to determine the structure and thickness of the CVD grown graphene film. The films are found to be flat and homogeneous and can be suspended on the grid, indicative of good mechanical properties (Fig. 2a) [17]. The edges of the suspended film always fold back, allowing for a cross sectional view of the film. The observation of these edges by TEM provides an accurate way to measure the number of layers at multiple locations on the film [18]. To confirm the exact number of layers in the sample, we observed their folded regions by using an electron beam parallel to them. The folded region exhibits only one dark line, suggesting monolayer graphene structure (Fig. 2b) [19]. Typical patterns with sixfold symmetry are observed by SAED from the region marked with the white circle in Fig. 2a, indicating the single-crystalline nature of the observed domain (Fig. 2c) [20]. For further quantitative analysis of diffraction patterns, we labeled the peaks with Bravais-Miller indices. As shown in Fig. 2d, the inner peaks ($0\overset{-}{1}10$) and ($\overset{-}{1}010$) are more intense than the outer ones ($1\overset{-}{2}10$) and ($\overset{-}{2}110$), further confirming the monolayer nature of the graphene [20, 21].

**Fig 3:**
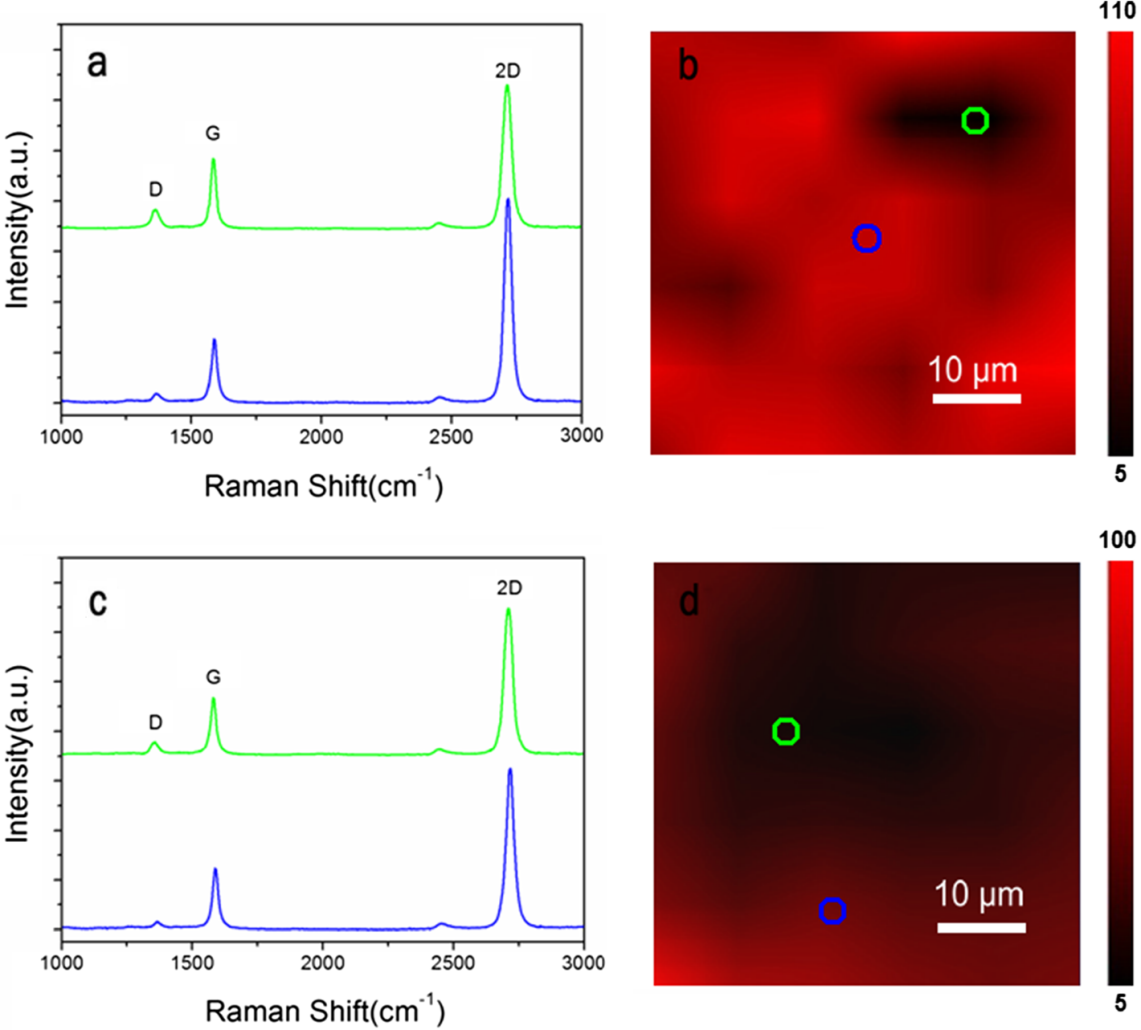
Raman spectroscopy analysis of graphene on BMN film. **a** Raman spectra from *marked spots* with corresponding colored circles in (**b**). **b** Raman maps of graphene from the *top* graphene electrode. Scale bars, 10 μm. **c** Raman spectra from marked spots with corresponding *colored circles* in (**d**). **d** Raman maps from the *bottom* graphene electrode. Scale bars, 10 μm

To identify the quality and uniformity of graphene, the Raman spectra were collected from graphene on the top and bottom of BMN film in the devices. Figure 3a shows the Raman spectra from graphene as the top of the BMN film. The G and 2D peaks of graphene are clearly seen at ~1590 and ~2695 cm^−1^, respectively [22]. The G peak is caused by the first-order scattering of the in-plane optical phonon E_2g_ mode, and the 2D band is the result of a second-order process involving two phonons with opposite momentum [22, 23]. The Raman intensity ratio *I*_2D_/*I*_G_ is larger than 2.0 and the full width at half maximum of 2D peak is ~30 cm^−1^, which can be interpreted as the structure of monolayer graphene [24, 25]. The D peak at ~1350 cm^−1^ corresponds to the breathing mode of sp^2^ hexagonal carbon but only gets activated by a defect with the help of a double resonance in the inter-valley scattering [26]. In order to evaluate the uniformity and defect density level of graphene, we also map the spatial variation of the intensity ratios of *I*_G_/*I*_D_ in an area of 50 × 50 μm^2^ (Fig. 3b). The map shows different color contrast from dark red to bright red, reflecting diversity of defect density in graphene. For the spectrum from the bright red region (blue circle in Fig. 3b), the defect-related D band was negligible (blue curve in Fig. 3a). For the spectrum from the dark red region (marked in green circle), though the defects related D bands are increased, the value is still very weak (green curve Fig. 3a). These results demonstrate that the graphene on the BMN film as the top electrode are of high quality.

Figure 2c shows the Raman spectra of graphene as the bottom electrode on BMN film. The spectra also show the typical feature of graphene. The blue and green spectral curves respectively correspond to the blue and green circle marked in the Raman mapping in Fig. 2d. The different values of *I*_G_/*I*_D_ in the mapping from the red and dark red regions of the map show the diversity of disruptions. However, though the dark red region was even larger than bright red one, the defect-related D bands collected from the dark red region are very weak. This fact indicates the graphene structure was only slightly affected by sputtering effect of LMBE. The less damage can be attributed to the high gas press and low laser fluence used in BMN film growth. In the Raman mapping experiment, we also mapped the spatial variation of the intensity ratios of Raman 2D peak to Raman G peak (*I*_2D_/*I*_G_) of graphene on BMN film (Additional file 1: Figure S1). The map shows that almost all the regions have 2D peak intensity twice larger than that of the G peak, indicating that the graphene are monolayer structure and highly continuous on BMN film [24, 25].

### Comparisons of BMN Films Grown on Graphene With That Grown on Au Substrate

**Fig 4:**
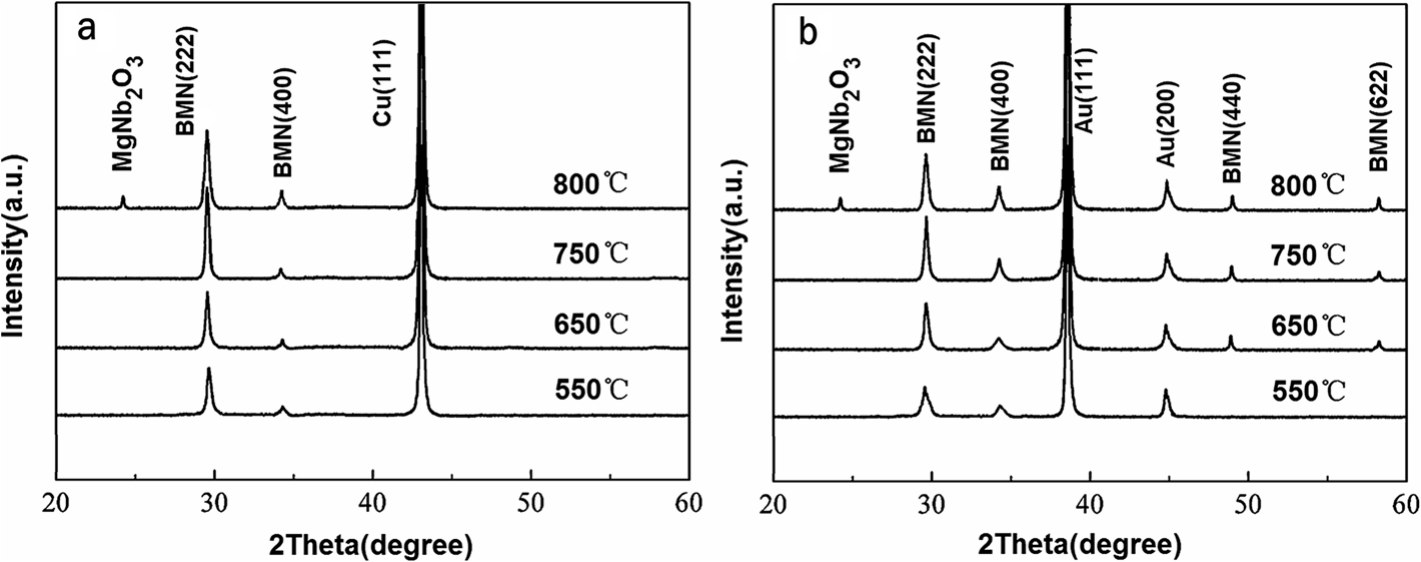
Comparison of XRD patterns of the BMN films grown on graphene/Cu and Au substrates. **a** XRD patterns of BMN films grown on graphene at growth temperature of 550–800 °C. **b** XRD patterns of BMN films grown on Au substrates at the corresponding temperature of 550–800 °C

Figure 4a shows XRD patterns of the BMN films grown on graphene/Cu substrates at growth temperature of 550–800 °C. At growth temperature of 550 °C, the BMN thin films show cubic pyrochlore phase with a main peak (222) at 2*θ* = 29.3°. As growth temperature increases from 650 to 750 °C, the intensities of diffraction peaks strengthen, indicating the improvement of crystallinity. For the films grown at 800 °C, the main peak (222) decreases and the secondary phase MgNb_2_O_6_ (JCPDS card number 33–0857) is detected [27]. The emergence of MgNb_2_O_6_ can be ascribed to the volatilization of Bi element at high temperature. The lattice constant of the BMN films calculated from the XRD patterns is about 10.529 Å, which is similar to the value of BMN thin films on Pt-coated sapphire substrates [28]. Figure 4b shows XRD patterns of BMN films grown on Au substrates at the corresponding temperature of 550–800 °C. Compared to graphene/Cu substrates, more peaks such as (440) and (622) are observed and the intensity of the main peak is lower at the corresponding temperature. The intensity ratios of the main peak to the second high peak are listed in Table 1. As shown in Additional file 2: Table S1, a larger intensity ratio is acquired from the BMN film grown on graphene at the same growth temperature. These results indicate that the BMN films grown on graphene have better (222)-oriented texture and higher crystallinity. Since the pure cubic pyrochlore structure with a preferred (222) orientation is regarded to be essential for large tunability, the BMN films grown on graphene are expected to acquire better tunability. The full width at half maximum (FWHM) of the main peak (222) of BMN films grown at 750 °C for different substrates is also compared (Additional file 3: Figure S2). For the film grown on graphene, its FWHM is ~23°, which is much smaller than that of the film grown on Au substrate. The high crystallinity of BMN on graphene can be explained by the distinctive properties of graphene as graphene layer has a van der Waals interface, which can drastically relax the lattice matching condition and improve heteroepitaxial growth [29–31].

**Table 1:** Dielectric properties and transmittances of BMN films fabricated at different growth temperatures

Temperature (°C)	550	650	750	800
Transmittance at 550 nm (%)	80.3	85.7	91.1	89.3
Tunability at 1 MV/cm (%)	13.5	26.1	40.7	32.1
Maximum dielectric constant	68	88	113	106
Loss tangent at 0–1 MV/cm	0.047–0.058	0.039–0.044	0.032–0.036	0.064–0.077

**Fig 5:**
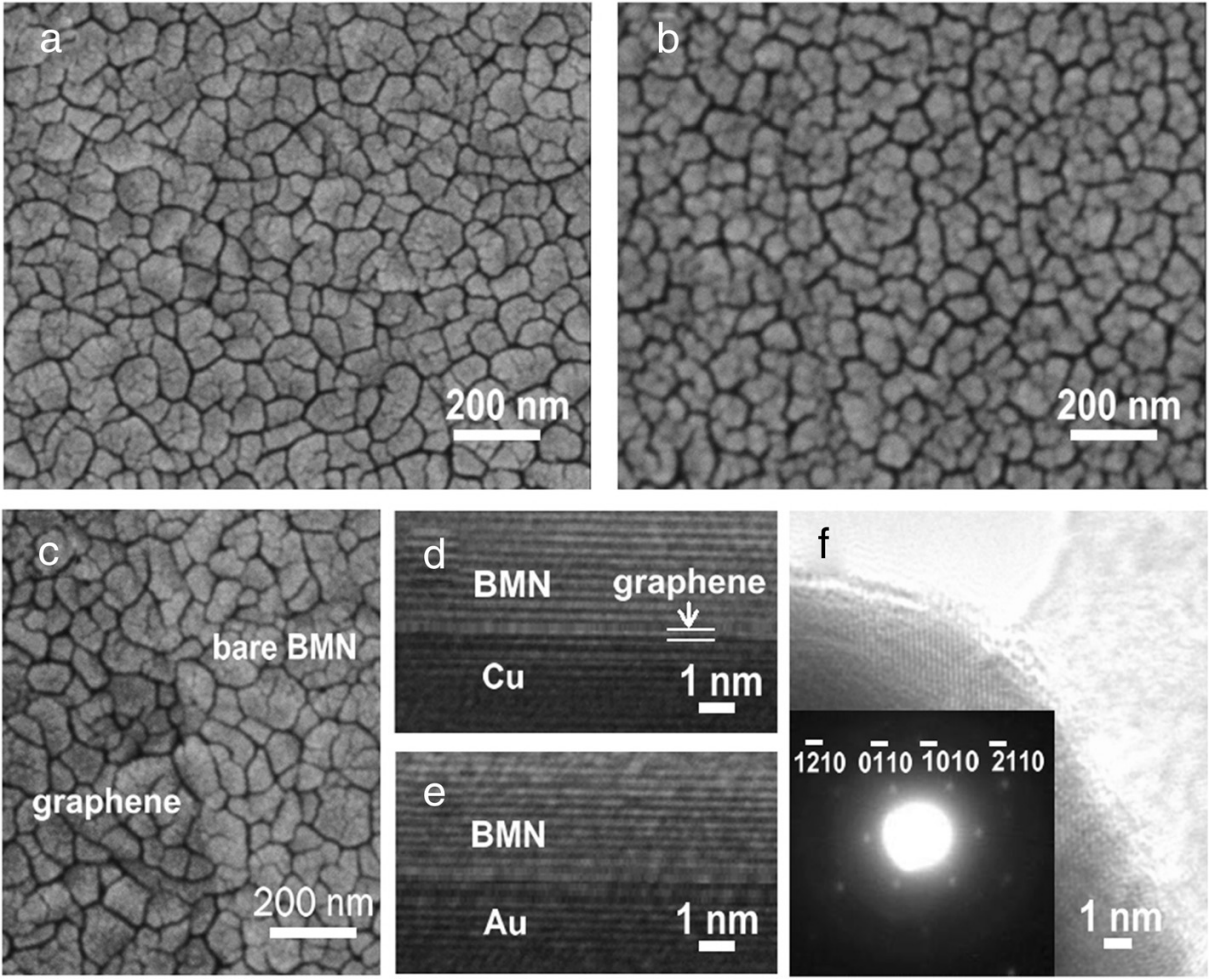
SEM image of BMN film grown on graphene/Cu (**a**) and Au substrate (**b**). c A piece of graphene on BMN film. **d** Cross-section HRTEM image of the BMN film on graphene/Cu. (**e**) Cross-section HRTEM image of the BMN film on Au substrate. **f** HRTEM image of the graphene-covered BMN grain, the *inset* shows the hexagonal symmetry lattice points of graphene

Figure 5 shows the surface morphology of BMN film grown on graphene/Cu and Au substrate, respectively. For the BMN film grown on graphene, it displays a uniform surface formed by many polyhedron grains, which is consistent with the crystal phase shown by XRD patterns in Fig. 4a. The grains have a large size of 100–200 nm, and the boundaries among them are clear, indicative of the high crystallinity of BMN film. Figure 5b shows the surface of BMN film grown on Au substrate. The film shows a similar grain size to that on graphene. However, the grain is not as smooth as that on graphene. Compared to the film on Au substrate, the one grown on graphene has denser surface and larger grain size. It is indicated that the crystallinity of thin films grown on Au substrate has not been well developed compared to that grown on graphene. These results demonstrate that the crystallization is improved by adding a graphene layer. The surface morphologies of BMN films grown on graphene/Cu at different temperatures are also compared (Additional file 4: Figure S3). By increasing the growth temperature from 550 to 800 °C, the average surface grain size rises and the crystallization is promoted. Compared to lower growth temperatures, the films grown at 750 and 800 °C have denser surface and larger grain size. Figure 5c shows the SEM image of a piece of graphene on BMN film. The graphene-veiled BMN film shows a darker color compared to the bare film, forming a clear boundary with the uncovered region. Due to the atom-thick nature of graphene, the BMN surface morphology can also be clearly seen through the graphene layer. The graphene membrane follows the curvature of the underlying BMN film by an intimate contact with the BMN film. No cracks or tears of graphene are observed on the surface of the BMN film, indicating that the graphene membranes are well transferred [13]. Figure 5d, e shows a cross-section HRTEM image of the BMN film on graphene/Cu and Au substrate, respectively. The lattice planes of BMN, Au, and Cu can be clearly seen nearby the interface region. Compared to the interface between BMN film and Au, BMN film on graphene/Cu shows a clearer interface. This fact directly conform that the van der Waals surface of graphene is more suitable for the BMN film growth. Figure 5f shows the HRTEM image of the graphene-covered BMN grain. The clear BMN parallel lattice planes below graphene indicate intimate contact of graphene over BMN surface [13, 26]. Also, the hexagonal symmetry lattice points in the reciprocal space of graphene are observed. The patterns show a typical monolayer structure with a ratio of *I*_{1100}_/*I*_{2110}_ > 1.

**Fig 6:**
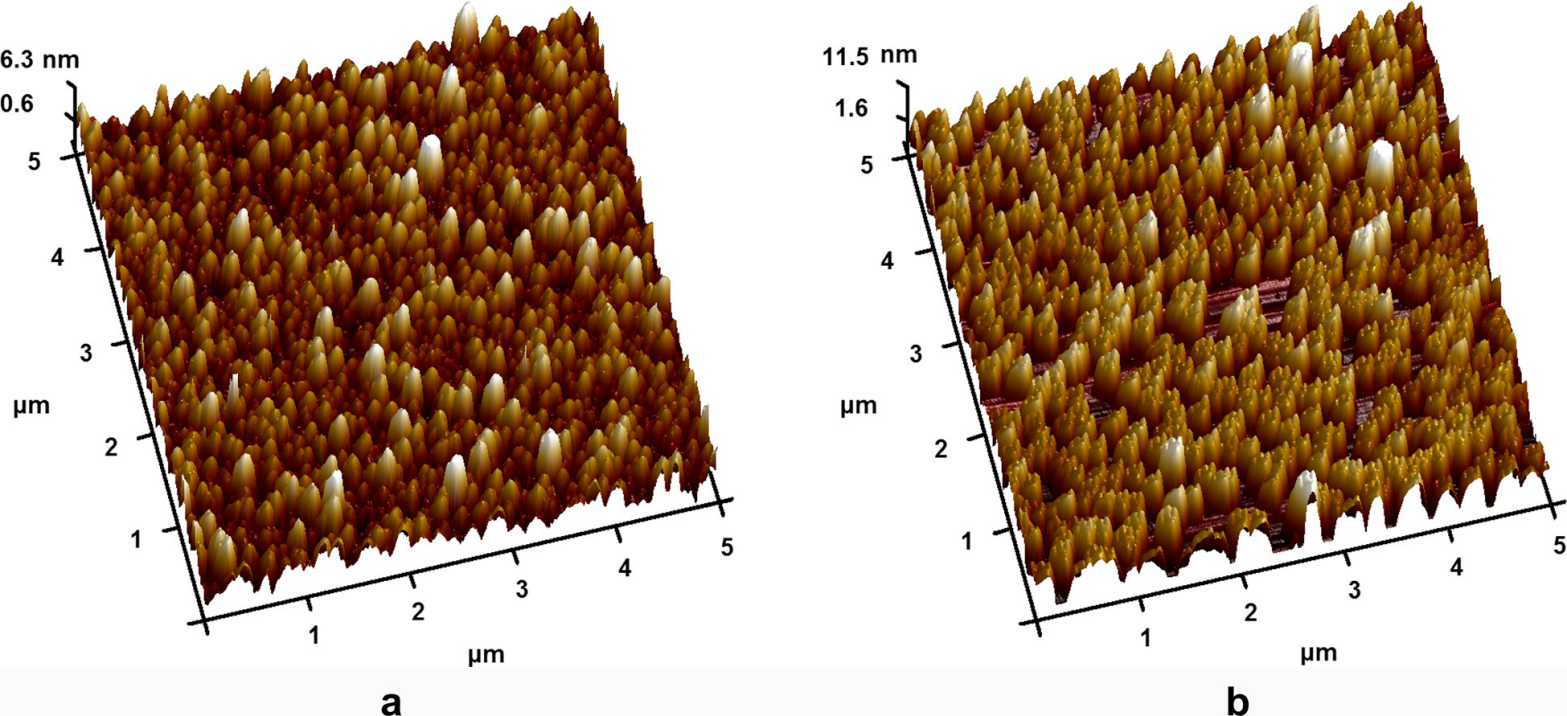
AFM image of BMN film grown on graphene/Cu (**a**) and Au substrate (**b**)

Surface roughness of the BMN films was evaluated by AFM. Figure 6a, b shows the AFM surface images of BMN films grown on graphene and Au substrate at 750 °C, respectively. The surface of the BMN films deposited on graphene is smooth with average roughness of 2.66 nm. For the BMN films grown on Au substrate, its surface is relatively rough with average roughness of 6.38 nm. The difference of the roughness may also attributed to intrinsic properties of the grown substrates. The graphene has a van der Waals surface, which is suitable for heteroepitaxial growth. For the BMN films grown on graphene substrate, the average surface roughness of BMN films grown at 550, 650, 750, and 800 °C is 1.71, 2.31, 2.66, and 3.59 nm, respectively (Additional file 5: Figure S4). Though the average grain size is much increased with the growth temperature (SEM in Additional file 4: Figure S3), the surface roughness of the film is only slightly increased. The low surface roughness can be attributed to the slow growing rate of the film. The slow growing pattern is favor for stress release and smooth surface.

### Performance of Flexible and Transparent Tunable Capacitors

**Fig 7:**
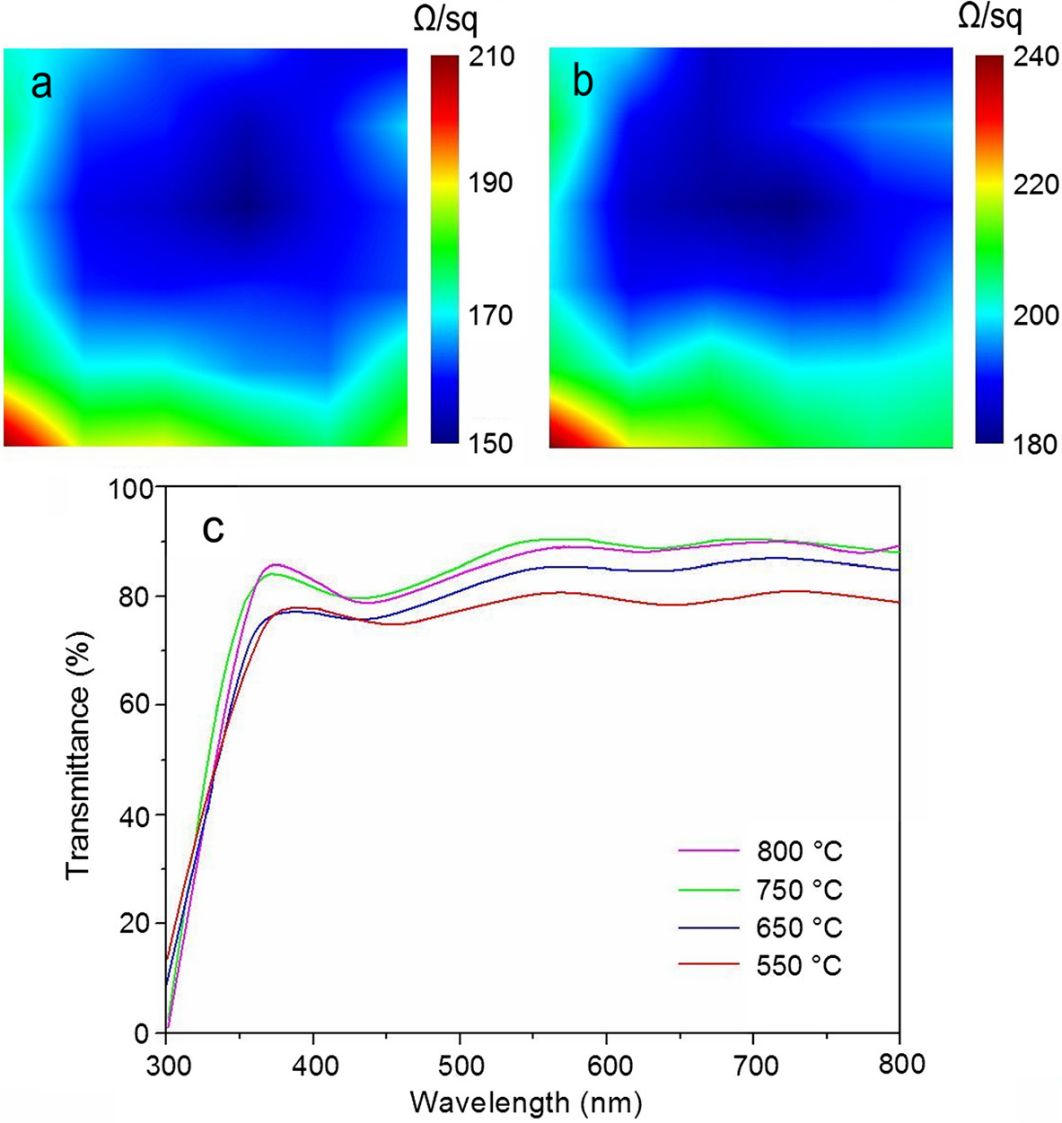
The spatial distributions of sheet resistances tested on a 3 × 3 cm^2^ on *top* graphene electrode (**a**) and *bottom* graphene electrode (**b**). **c** Optical transmittance of the tunable capacitors with the BMN films grown at different temperatures 550–800 °C

As a kind of electrode materials, the electrical properties of graphene are an important parameter in device fabrication. The spatial distributions of sheet resistances tested on a 30 × 30 mm graphene on top and bottom of the BMN film are shown in Fig. 7a, b, respectively. The data of sheet resistances were recorded at every 2 mm over 30 × 30 mm area and the total 225 points was measured for both graphene on top and bottom of the BMN film. The color mappings of the spatial distributions of sheet resistances were obtained by MATLAB software from the measured data. For the top graphene electrode, its sheet resistance is in the range of 150–210 Ω/sq. For the bottom graphene electrode, its sheet resistance is a little higher with the value of 180–240 Ω/sq. The increase of resistance can be attributed to the sputtering effect during the LMBE process. Though the low laser fluence and high deposition pressure are adopted, the structure of graphene will also inevitably be affected by the sputtering effect, especially at the early growth stage of BMN film. For both graphene electrodes, the edges of the samples have larger sheet resistance compared to the middle. This phenomenon might be associated with the edge stress of the BMN film. For the capacitor fabrication, we choose the middle section as the top and the bottom electrodes, which have uniform sheet resistance and suitable for electrode. The transparency is also an important parameter for transparent tunable capacitors. Figure 7c shows the optical transmittances of the tunable capacitors with the structure of graphene-BMN-films in the range of 400–800 nm. The oscillation in the transmittance curve is caused by the interferences at the air—and substrate-film interfaces [32]. The sharp fall in transmission at the shorter wavelength is due to the fundamental absorption of the BMN film [32]. At the wavelength of 550 nm, the transmittance of the BMN film grown at 550 °C is ~80.3 %. The transmittance increases significantly with the increase of grown temperature. For the samples fabricated at 650, 750, and 800 °C, their transmittance at 550 nm is ~85.7, ~89.3, and ~91.1 %, respectively. The high correlation between transmittance and grown temperature can be explained by the fact that the BMN films grown at higher temperature have better perovskite crystallinity, thus reducing the grain boundary scattering and increasing the optical transmittance [33]. Optical transmittance data reveal that the capacitors have satisfactory transparency for integration with other optoelectronic device.

**Fig 8:**
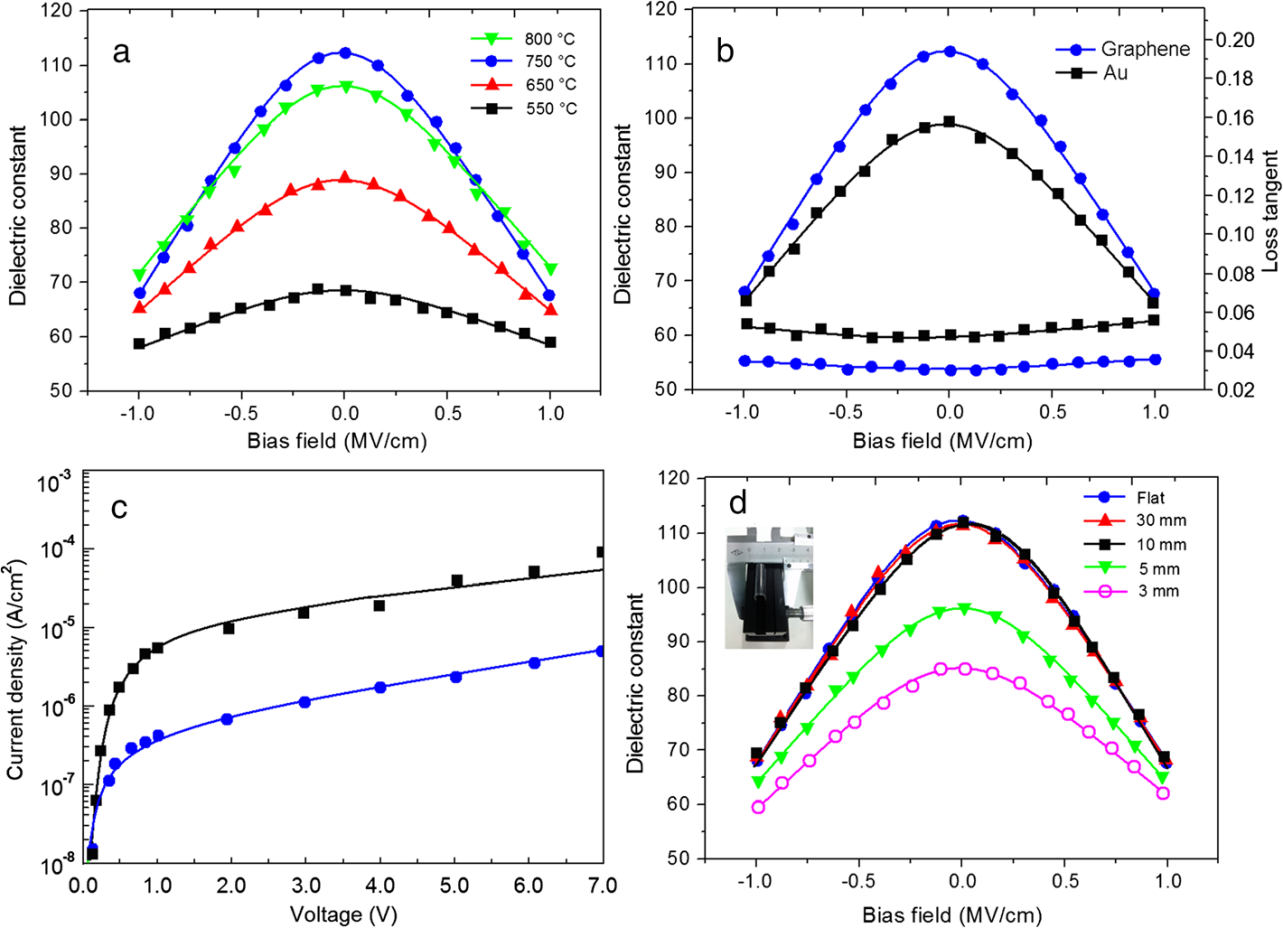
**a** The bias-field dependence of the dielectric properties of BMN films at 550–800 °C. **b** The tunability and loss tangent of BMN film grown on graphene and Au at 750 °C. **c** The leakage current density versus electrical field for the BMN films grown on graphene and Au substrate. **d** Measured dielectric constant versus bias-field at the flat and bending conditions with curvature radii from 30 to 3 mm. The *top left inset* presents the homemade mechanical device for the bending test

Figure 8a shows the tunability of the capacitor measured at 1 MHz. The dielectric constant *ε* is calculated from the capacitance *C*:

1 $$ \varepsilon =\frac{C\times d}{\varepsilon_0\times A} $$

where *d* is the BMN film thickness, *ε*_0_ is the dielectric constant in vacuum, and *A* is the capacitor area. The maximum dielectric constant reaches about 113 for the sample grown at 750 °C. The tunability of the thin films is defined as [34–36]:

2 $$ \eta =\frac{\varepsilon (0)-\varepsilon (v)}{\varepsilon (0)} $$

where *ε*(0) and *ε*(v) are the dielectric constant at zero and a certain bias field, respectively. For the films grown at 550 °C, the tunability of the dielectric constant is very low of ~13.5 % at a bias field of 1.0 MV/cm, which is consistent with their relative poor crystallized phase. The tunability of the film increases with growing temperature from 550 to 750 °C, possibly due to the enhancing crystallization of the films. For the films grown at 750 °C, the maximum tenability of ~40.7 % is achieved at a bias field of 1.0 MV/cm. Compared to the BMN thin film grown at 750 °C, the sample grown at 800 °C shows a decrease of tunability, which can be ascribed to the appearance of additional phase of MgNb_2_O_6_ as illustrated in XRD patterns in Fig. 4a. The MgNb_2_O_6_ phase has a lower *ε* of ~19.9 and may also disorders the cubic pyrochlore structure of BMN, reducing the polarization of BMN [37]. The loss tangent of the films grown at 550–800 °C was also measured as shown in the Additional file 6: Figure S5. The loss tangent was decreased with the increase when the temperature was lower than 750 °C. While when the growth temperature was increased to 800 °C, the loss tangent of the film was sharp increased. The sharp increase of loss tangent can also be attributed to the additional phase of MgNb_2_O_6_ as shown in Fig. 4a. For all cases, the loss tangent of the films goes up with the increasing of the applied voltage. The loss tangent as a function of bias field can be explained by the fact that high bias field leads to higher leakage, which causes a higher dielectric loss. As the tunable capacitor was transparent, the bias-field dependence of the dielectric properties and transmittances of BMN films fabricated at different growth temperatures was summarized in Table 1 according to the results of Fig. 7c and Fig. 8a and Additional file 6: Figure S5. As shown in Table 1, the film grown at 750 °C shows the highest transmittance (91.1 %), tunability (40.7), maximum dielectric constant (113), and the lowest loss tangent (0.032–0.035), indicating the optimal growth temperature for BMN film. Figure 8b compares the tunability and loss tangent of BMN film grown on graphene with that on Au substrate at 750 °C. For comparison, graphene was also used as electrode for the BMN film grown on Au substrate and the Au film with 0.2 mm in diameter was also patterned on the top of graphene. For sample grown on Au, the tunability is about 33.6 %, 7.1 % lower than that of the sample grown on graphene substrate. For loss tangent comparison, the loss tangents of the BMN film grown on graphene are lower than that of BMN film grown on Au substrate at various bias-fields. The high tunability and relative low loss tangent of the BMN film grown on graphene substrate can be explained by its better (222)-oriented cubic pyrochlore structure, which is regarded to be essential for large tenability and low loss tangent [27]. Also, the dielectric properties of the capacitors are compared with those of the other BMN-based capacitors, in terms of the maximum dielectric constant, tunability, and loss tangent as shown in Table 2. Compared with results of the previous reports about BMN films grown on the Pt [28], Au [38], and ITO [32] substrates, the BMN film grown on graphene has larger dielectric constant (compared with the film with similar thickness) and larger tunability (even at a minor bias-field of 1 MV/cm). The large dielectric constant and tunability can both be attributed to the improved crystallization of BMN induced by unique structure of graphene as discussed in the XRD and SEM sections. However, compared with loss tangent from other reports, the loss tangent of capacitors based on BMN film on graphene was much larger. By considering that the BMN film grown on Au substrate in our contrast experiment also has large loss tangent (Fig. 8b), the large loss tangent of this work should be associated with the relatively large resistance of graphene, as the loss tangent has been known to be inversely proportional to the electrode resistance or any interfacial resistance when the measurement frequency was at 1 MHz [34]. Figure 8c shows the leakage current density versus electrical field for the BMN film grown on graphene and on Au substrates. The leakage current density of BMN film varies exponentially with electric field. This phenomenon is in accordance with Poole-Frenkel (PF) conduction and Schottky emission theories [39–41]. The leakage current density of the BMN film grown on Au is at ~4.5 × 10^−5^ A/cm^2^ at a bias voltage of 7.0 V. In contrast, the sample grown on graphene shows a low leakage current density of ~4.0 × 10^−6^ A/cm^2^ at a bias voltage of 7.0 V. The difference of the leakage current is thought to be associated with both surface roughness and crystallinity of the BMN films. For the film grown at Au, its surface has a relatively high roughness of 6.38 nm. The local electric field will vary from place to place due to the fluctuation of the film surface. At the valley, the electric field is larger than that at the peak, which increases the leakage current density of film [42]. Moreover, the BMN film grown on graphene is much denser than that grown on Au, which also leads to a lower leakage current density. The same conclusion can also be obtained in the comparison of leakage current density for the samples grown at different temperatures in the Additional file 7: Figure S6. These results demonstrate that the graphene layer effectively improves the BMN film quality and reduces leakage current density. Considering that BMN films, graphene electrodes are flexible, these kinds of tunable capacitors thus can work at bending conditions. For measurement, the capacitors were attached onto a ~50-μm-thick PET substrate. The top left inset in Fig. 8d presents the homemade mechanical device for the bending test. After clamping the patterned Au on graphene by two fixtures connected to the capacitance measurement system, the PET substrate with the film capacitor can be compressed to a specific radius of curvature. Figure 8d shows the dielectric constant at the flat and bending conditions with curvature radii from 30 to 3 mm. For each curvature radius, the film capacitor was all bent over 50 times and the data error was less than 5 %. Compared to the flat condition, the dielectric response is nearly invariant until the bending radius decreases to about 10 mm, indicating that the capacitors can work stably in very high bending status. For the curvature radii below 10 mm, both tunability and dielectric constant of the capacitors obviously decrease. The decreased tunability can be attributed to the large tensile stress, which can reduce the dielectric polarization as reported in the previous literature [43, 44]. The bending test results suggest that the graphene-based capacitors have excellent mechanical stability, which are expected to be used as transparent, flexible, and tunable capacitors.

**Table 2:** Comparison of dielectric properties of BMN thin films from this research with those taken from other references

Reference	Maximum dielectric constant	Tenability (%)	Loss tangent	Growth substrate	Film thickness (nm)
Jiang [28]	86	39 (1.6 MV/cm)	0.005 (100 KHz)	Pt	250
Ning [38]	135	31.3 (1 MV/cm)	0.002 (1 MHz)	Au	400
Yu [32]	100	29 (1.8 MV/cm)	–	ITO	220
This work	113	40.7 (1 MV/cm)	0.032 (1 MHz)	Graphene	220

## Conclusions

We fabricated a kind of electric field tunable transparent and flexible capacitor by using graphene as electrodes. The graphene film was grown by CVD method, and the BMN thin films were fabricated on graphene by using LMBE technology. The comparison of BMN films grown on graphene with Au substrates shows that the graphene can obviously improve BMN film quality in terms of crystallinity, surface morphology, leakage current, and loss tangent. By using transfer method, we fabricated flexible and transparent capacitors with the structure of graphene-BMN-graphene. The capacitors show the largest dielectric constant of 113 and the largest dielectric tunability of ~40.7 % at 1.0 MV/cm and can work stably in the high bending condition with curvature radii as low as 10 mm. This film capacitor has a high optical transparency of ~90 % in the visible light region, making tunable capacitor highly promising for integration into flexible optoelectronic devices.

## Additional Files

Additional file 1: Figure S1. The Raman spectra (a) and Raman mapping (b) of graphene on BMN film. The Raman map shows the spatial variation of the intensity ratios of Raman 2D peak to Raman G peak (*I*_2D_/*I*_G_) for graphene on BMN film. The maps show that 2D peak intensity twice larger than that of the G peak, indicating that the graphene are monolayer structure and highly continuous on BMN film. Additional file 2: Table S1. The intensity ratio of main peak (222) to second high peak (400). Additional file 3: Figure S2. The FWHM comparison of main peak (222) of BMN films grown on graphene with that grown on Au substrate at 750 °C. (a) The FWHM of peak (222) of BMN films grown on graphene. (b) The FWHM of peak (222) of BMN films grown on Au substrate. For the BMN film grown on graphene, its FWHM value is much smaller, indicative of the better crystallinity. Additional file 4: Figure S3. The surface morphologies of BMN film grown on graphene/Cu at different temperatures of 550–800 °C. (a) 550, (b) 650, (c) 750, and (d) 800 °C. By increasing the growth temperature from 550 to 800 °C, the average surface grain size rise and the crystallization is promoted. Compared with lower temperatures, the films grown at 750 and 800 °C have denser surface and larger grain size. Additional file 5: Figure S4. AFM images of BMN films grown on graphene substrate grown at different temperatures. (a) 550, (b) 650, (c) 750, and (d) 800 °C. The surface roughness of BMN films grown at 550, 650, 750 and 800 °C were 1.71, 2.31, 2.66 and 3.59 nm, respectively. Though the grain size is much increased with the growth temperature, the surface roughness of the film is only slightly increased. The low surface roughness can be explained by the slow growing rate of film. The slow growing pattern is favor for stress release and a smooth surface. Additional file 6: Figure S5. Loss tangent of BMN films grown on graphene substrate at different temperature. (a) 550, (b) 650, (c) 750, and (d) 800 °C. With the increase of temperature from 550 to 750 °C, the loss tangent of the samples is significantly decreased, which can be attributed to the improvement of crystal quality. Abrupt increase of loss tangent of samples grown at 800 °C can be ascribed to the appearance of additional phase as illustrated in XRD patterns in Fig. 4a. Additional file 7: Figure S6. Leakage current density of BMN films grown on graphene substrate at different temperatures. (a) 550, (b) 650, (c) 750, and (d) 800 °C. With the increase of temperature from 550 to 750 °C, the leakage current density of the samples is gradually decreased at the same voltage, which can be also attributed to the improvement of crystal quality. The samples grown at higher temperature tend to have denser surface, which is beneficial to reduce current density. Abrupt increase of leakage current density of samples grown at 800 °C is thought to be associated with additional phase of MgNb_2_O_6_ as illustrated in XRD patterns in Fig. 4a.
