# Supplementary figures and images for: Graphene-Based Flexible and Transparent Tunable Capacitors
Source: Nanoscale Res Lett. 2015 Jul 3;10:279. doi: 10.1186/s11671-015-0974-4 (PMC4489973; doi:10.1186/s11671-015-0974-4)

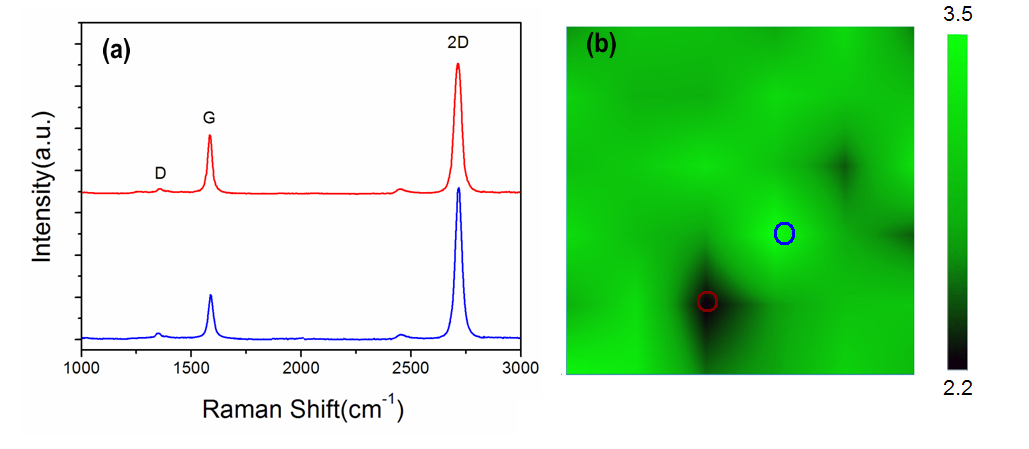

Supplement: Additional file 1: Figure S1. — The Raman spectra (a) and Raman mapping (b) of graphene on BMN film. The Raman map shows the spatial variation of the intensity ratios of Raman 2D peak to Raman G peak (I 2D/I G) for graphene on BMN film. The maps show that 2D peak intensity twice larger than that of the G peak, indicating that the graphene are monolayer structure and highly continuous on BMN film. [file 11671_2015_974_MOESM1_ESM.tiff]

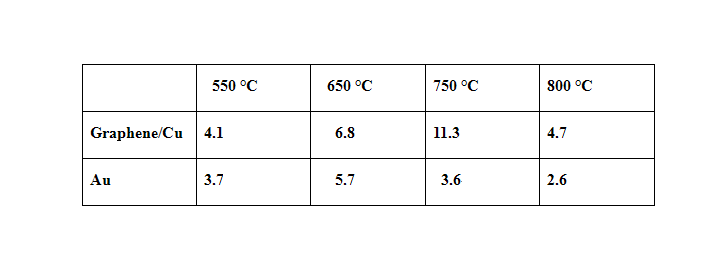

Supplement: Additional file 2: Table S1. — The intensity ratio of main peak (222) to second high peak (400). [file 11671_2015_974_MOESM2_ESM.tiff]

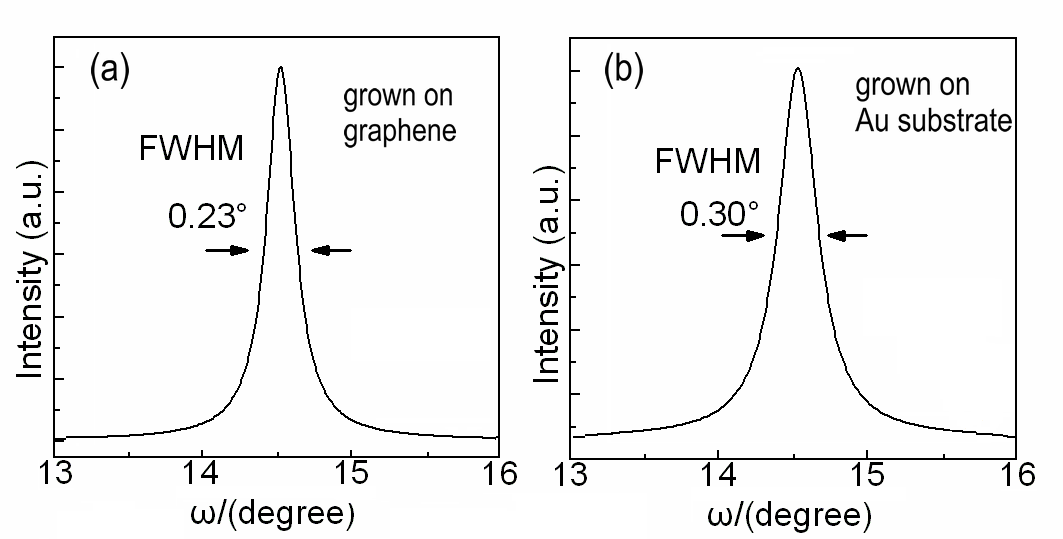

Supplement: Additional file 3: Figure S2. — The FWHM comparison of main peak (222) of BMN films grown on graphene with that grown on Au substrate at 750 °C. (a) The FWHM of peak (222) of BMN films grown on graphene. (b) The FWHM of peak (222) of BMN films grown on Au substrate. For the BMN film grown on graphene, its FWHM value is much smaller, indicative of the better crystallinity. [file 11671_2015_974_MOESM3_ESM.tiff]

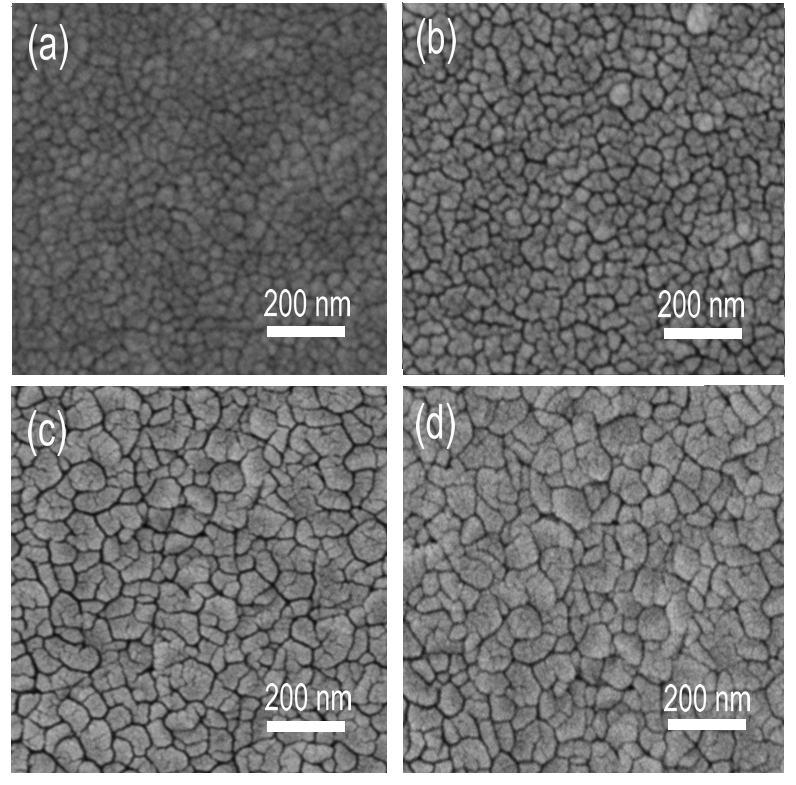

Supplement: Additional file 4: Figure S3. — The surface morphologies of BMN film grown on graphene/Cu at different temperatures of 550–800 °C. (a) 550, (b) 650, (c) 750, and (d) 800 °C. By increasing the growth temperature from 550 to 800 °C, the average surface grain size rise and the crystallization is promoted. Compared with lower temperatures, the films grown at 750 and 800 °C have denser surface and larger grain size. [file 11671_2015_974_MOESM4_ESM.tiff]

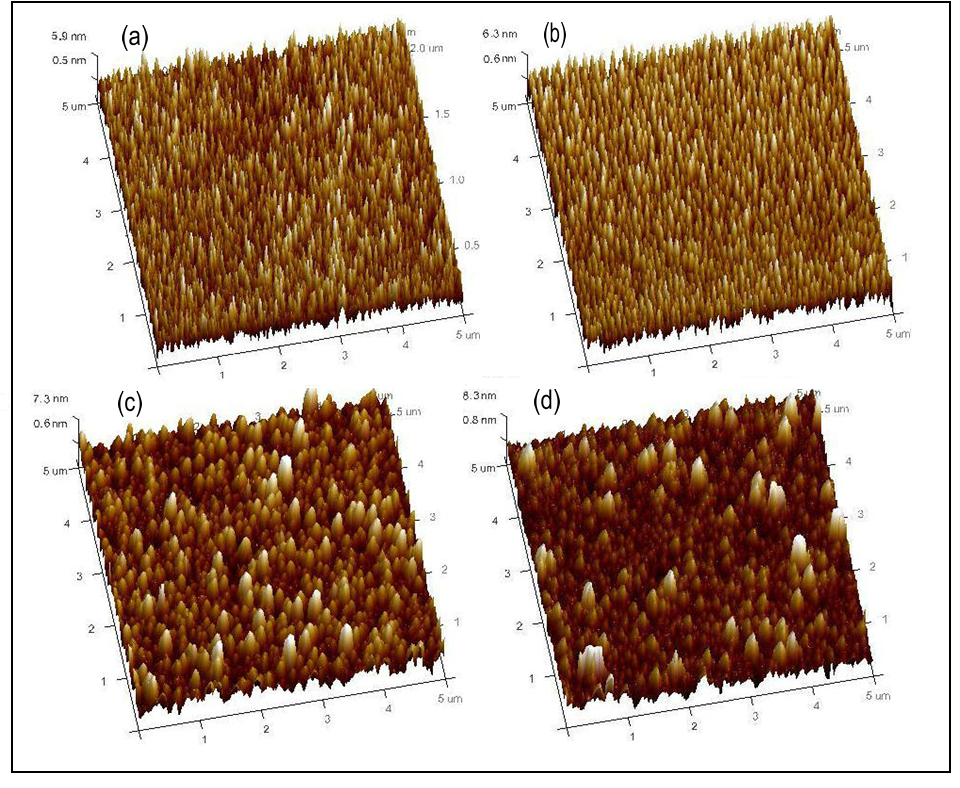

Supplement: Additional file 5: Figure S4. — AFM images of BMN films grown on graphene substrate grown at different temperatures. (a) 550, (b) 650, (c) 750, and (d) 800 °C. The surface roughness of BMN films grown at 550, 650, 750 and 800 °C were 1.71, 2.31, 2.66 and 3.59 nm, respectively. Though the grain size is much increased with the growth temperature, the surface roughness of the film is only slightly increased. The low surface roughness can be explained by the slow growing rate of film. The slow growing pattern is favor for stress release and a smooth surface. [file 11671_2015_974_MOESM5_ESM.tiff]

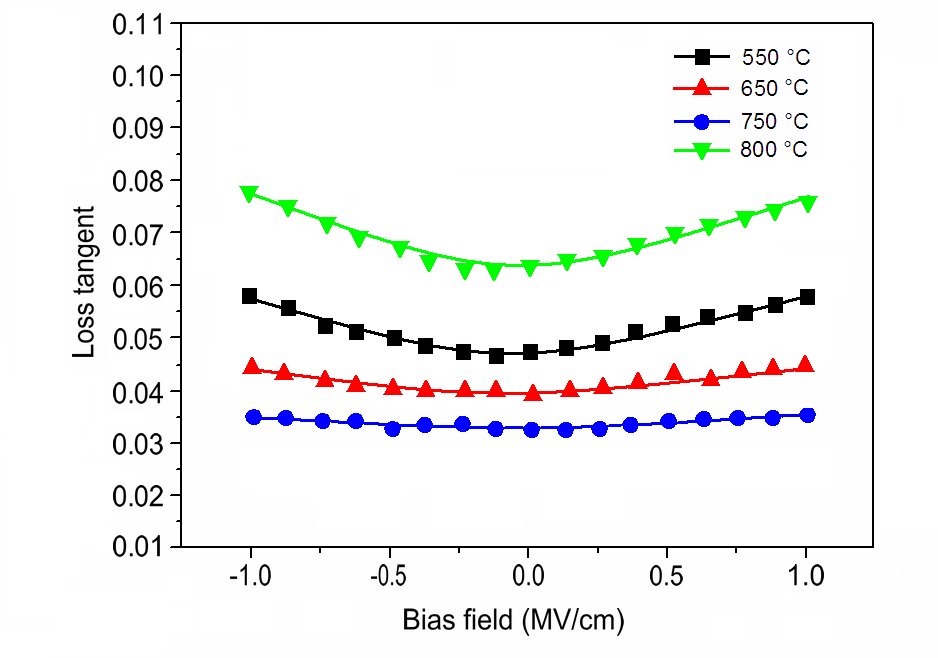

Supplement: Additional file 6: Figure S5. — Loss tangent of BMN films grown on graphene substrate at different temperature. (a) 550, (b) 650, (c) 750, and (d) 800 °C. With the increase of temperature from 550 to 750 °C, the loss tangent of the samples is significantly decreased, which can be attributed to the improvement of crystal quality. Abrupt increase of loss tangent of samples grown at 800 °C can be ascribed to the appearance of additional phase as illustrated in XRD patterns in Fig. 4a. [file 11671_2015_974_MOESM6_ESM.tiff]

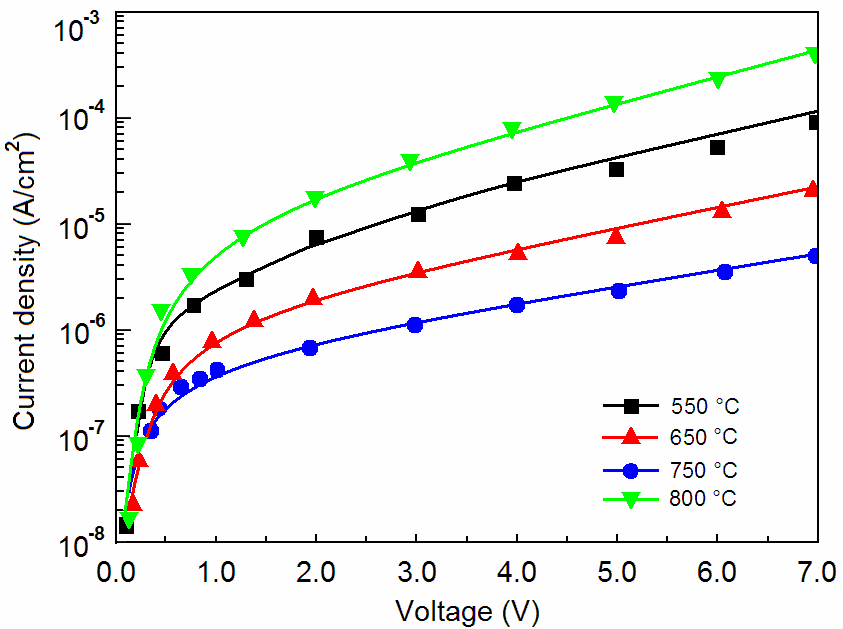

Supplement: Additional file 7: Figure S6. — Leakage current density of BMN films grown on graphene substrate at different temperatures. (a) 550, (b) 650, (c) 750, and (d) 800 °C. With the increase of temperature from 550 to 750 °C, the leakage current density of the samples is gradually decreased at the same voltage, which can be also attributed to the improvement of crystal quality. The samples grown at higher temperature tend to have denser surface, which is beneficial to reduce current density. Abrupt increase of leakage current density of samples grown at 800 °C is thought to be associated with additional phase of MgNb2O6 as illustrated in XRD patterns in Fig. 4a. [file 11671_2015_974_MOESM7_ESM.tiff]
